# OP34 Methodological Approach For Synthesizing Public Consultations To Inform Decision-Making In Health Technology Incorporation in Brazil: A Delphi study

**DOI:** 10.1017/S0266462325100925

**Published:** 2025-12-29

**Authors:** Viviane Karoline da Silva Carvalho, Jorge Otavio Maia Barreto, Everton Nunes da Silva

## Abstract

**Introduction:**

Public consultations (PCs) are widely used by health technology assessment (HTA) agencies, but there is no consensus on methodological procedures for analyzing PCs. We propose a method for analyzing and synthesizing PCs, offering an agile approach, systematic and transparent steps, reproducible aspects, and reliable analyses. The aim of this study was to validate the method.

**Methods:**

The Delphi technique was used to validate the proposed method in terms of face (applicability and relevance) and content validity (analysis results). The setting included the HTA processes conducted by the National Committee for Health Technology Incorporation (CONITEC). A group of 20 Brazilian experts was invited to participate, each of whom had experience in at least one of the following areas: HTA, social participation, IRaMuTeQ software, or qualitative research. Consensus was defined as at least 70 percent with “agree” or “strongly agree” responses or an interquartile range of one or less. Comments were summarized and categorized thematically. A pilot study was also conducted.

**Results:**

Fifteen experts participated in the face validity assessment and 14 in the content validity assessment. Both validations achieved over 80 percent consensus in the first round. In face validity, five methodological steps were validated, including corpus analysis, corpus preparation and organization, data mining using IRaMuTeQ software, systematization and interpretation, and synthesis of findings. In content validity, the results were deemed adequate, providing sufficient information to understand PCs. Although this method was used in the Brazilian HTA setting, it can be adapted by different countries. The method provides a way to organize data already collected in HTA PCs, contributing to processes already in use.

**Conclusions:**

This study validated a method for analyzing and systematizing HTA PCs—the first strategy of this kind. The method proposes a systematic and transparent approach to organizing data collected for HTA agencies. This initial strategy for systematizing PC analyses can support more participatory, agile, and transparent processes that include public perspectives, which can be a challenge in HTA.

